# Application of CRISPR/Cas9-mediated gene editing for abiotic stress management in crop plants

**DOI:** 10.3389/fpls.2023.1157678

**Published:** 2023-04-18

**Authors:** Manoj Kumar, Manas Ranjan Prusty, Manish K. Pandey, Prashant Kumar Singh, Abhishek Bohra, Baozhu Guo, Rajeev K. Varshney

**Affiliations:** ^1^ Institute of Plant Sciences, Agricultural Research Organization, Volcani Center, Rishon Lezion, Israel; ^2^ Institute for Cereal Crop Improvement, Plant Science, Tel Aviv University, Tel Aviv, Israel; ^3^ International Crops Research Institute for the Semi-Arid Tropics (ICRISAT), Hyderabad, India; ^4^ Department of Biotechnology, Mizoram University (A Central University), Pachhunga University College, Aizawl, India; ^5^ State Agricultural Biotechnology Centre, Centre for Crop and Food Innovation, Food Futures Institute, Murdoch University, Murdoch, WA, Australia; ^6^ Crop Genetics and Breeding Research Unit, United States Department of Agriculture-Agricultural Research Service (USDA-ARS), Tifton, GA, United States

**Keywords:** abiotic stress tolerance, base editing, CRISPR/Cas9, crop production, gene editing, prime editing

## Abstract

Abiotic stresses, including drought, salinity, cold, heat, and heavy metals, extensively reducing global agricultural production. Traditional breeding approaches and transgenic technology have been widely used to mitigate the risks of these environmental stresses. The discovery of engineered nucleases as genetic scissors to carry out precise manipulation in crop stress-responsive genes and associated molecular network has paved the way for sustainable management of abiotic stress conditions. In this context, the clustered regularly interspaced short palindromic repeat-Cas (CRISPR/Cas)-based gene-editing tool has revolutionized due to its simplicity, accessibility, adaptability, flexibility, and wide applicability. This system has great potential to build up crop varieties with enhanced tolerance against abiotic stresses. In this review, we summarize the latest findings on understanding the mechanism of abiotic stress response in plants and the application of CRISPR/Cas-mediated gene-editing system towards enhanced tolerance to a multitude of stresses including drought, salinity, cold, heat, and heavy metals. We provide mechanistic insights on the CRISPR/Cas9-based genome editing technology. We also discuss applications of evolving genome editing techniques such as prime editing and base editing, mutant library production, transgene free and multiplexing to rapidly deliver modern crop cultivars adapted to abiotic stress conditions.

## 1 Introduction

Abiotic stresses such as drought, heat, cold, salt, pesticides, metals and metalloids deteriorate agro-ecological conditions and hence, negatively impact upon agricultural production. The abiotic stress impairs with regular growth and development of plant plants, thus limiting their yield and quality of produce (Boyer, 1982; Pandey et al., 2017). Plants respond to abiotic stress conditions through changes in morphological, physiological, biochemical, and molecular levels (Wang et al., 2003). To feed the projected worldwide population of 9.7 billion by 2050, the global agricultural production needs to rise by at least 85% (Alexandratos and Bruinsma, 2012; Ray et al., 2013). Although conventional breeding approach has notably contributed to developing abiotic stress tolerance in crops, to achieve the projected gains in crop yields calls for implementing innovative technologies for rapid delivery of future crop cultivars equipped with stress adaptation traits.Also, the traditional way of breeding stress tolerant cultivars may take several years resulting in longer response time of crop researchers to evolving requirements of farming communities, growers and industry (Manavalan et al., 2009). Therefore, additional efficient and latest technologies with instant impacts are required to deal with these challenges (Driedonks et al., 2016). Genome editing tools enable precise changes in an organism’s DNA by introducing targeted mutation, insertion/deletion (indel), and specific sequence alteration *via* recruiting specific nucleases. During the past years, meganucleases (Puchta et al., 1993), transcription activator-like nucleases (TALENs) (Zhang et al., 2013) zinc-finger nucleases (ZFNs) (Zhang et al., 2010), and more recently, CRISPR–Cas9 (Jiang et al., 2013) have been developed and used for genome editing. The clustered regularly interspaced short palindromic repeats (CRISPR)/CRISPR-associated protein 9 (CRISPR/Cas9) has been demonstrated to be the most successful genome editing system across a wide range of organisms, including plants (Chen et al., 2019b) given its ability to induce desired mutations in efficient, cheaper, faster, and accurate manner (Chen et al., 2019b).

Additionally, this system can detect and cleave complementary DNA sequences in the genome. This application of targeted genome manipulation was inspired from a naturally occurring gene-editing system of bacteria to provide resistance against invading viruses. However, it is presently accepted as part of an adaptive defensive system that includes CAS enzymes associated with CRISPR/Cas9 (Barrangou et al., 2007). This technology may further help enable and promote the commercialization of resulting edited crop and food products to overcome societal apprehension s about involvement of ‘foreign DNAs’. However, few countries like Argentina have adopted genome-edited crops; conversely, several other countries are still debating this subject. Recently, CRISPR/Cas9 edited tomato, which contains higher amounts of γ-aminobutyric acid (GABA) than non-edited counterparts, has been commercialized in Japan’s market (Waltz, 2022). This technology is projected to go a long way toward enabling a comparatively painless acceptance of genome-edited crops in most countries.

CRISPR/Cas9 system has been applied in several plant species including model plants such as *Nicotiana benthamiana* (Li et al., 2013), *Nicotiana tabacum* (Donovan et al., 2020; Kumar et al., 2022) *Arabidopsis* (Li et al., 2013; Nekrasov et al., 2013) and in crop plants such as wheat (Shan et al., 2013), maize (Liang et al., 2014), rice (Miao et al., 2013; Shan et al., 2013) liverwort (Sugano et al., 2014), tomato (Brooks et al., 2014), potato (Wang et al., 2015), soybean (Jacobs et al., 2015), sweet orange (Jia and Nian, 2014), banana (Tripathi et al., 2019), pepper (Park et al., 2021), barley (Zeng et al., 2020; Garcia-Gimenez and Jobling, 2021), peanut (Yuan et al., 2019; Wei et al., 2021), foxtail millet (Lin et al., 2018), and sugarcane (Oz et al., 2021). Additionally, CRISPR/Cas9-based multiplexing by targeting multiple genes in a single organism has also been carried out successfully across a range of crop species such as wheat (Wang et al., 2018), rice (Miao et al., 2013), cotton (Gao et al., 2017) and maize (Char et al., 2017). Therefore, this technology has huge potential to produce genome-edited crop plants tolerant to multiple stresses by targeting numerous stress-sensitive genes concurrently in an elite high-yielding, but sensitive cultivar, and tolerance genes can also be overexpressed using CRISPR-mediated gene activation (Zafar et al., 2020). The genes implicated in stress-associated gene regulatory networks, signal transduction and metabolite production may be targeted *via* CRISPR/Cas9 technologies to develop stress-tolerant crop plants (Jain, 2015). Mushtaq et al. (2018) reported that the CRISPR/Cas-based gene-editing tool could efficiently target complex quantitative genes associated with abiotic stresses directly or indirectly. CRISPR/Cas9-mediated gene editing of various genes, including betaine aldehyde dehydrogenase (*OsBADH2*), mitogen-activated protein kinase (*OsMPK2*), stress/Abscisic acid (ABA)-activated protein kinase 2 (*SAPK2*), and phytoene desaturase (*OsPDS*) showed their implications for improving abiotic stress tolerance in rice (Shan et al., 2013; Lou et al., 2017). In plants, abiotic stress tolerance was improved through gene editing of ethylene responsible factor (ERF, a transcriptional factor) of the *AP2/ERF* superfamily (Debbarma et al., 2019).

Recently, Nascimento et al. (2023) discussed the role of CRISPR/Cas as an auxiliary tool in crop breeding programs targeting to grow additional modified cultivars to diverse abiotic factors. The review offers a broad and unbiased collection of relevant studies through a systematic search of databases on the application of CRISPR/Cas approach for abiotic stress tolerance (Nascimento et al., 2023). Similarly, Hossain et al. (2022) updated the concept, mechanism, and application of CRISPR-Cas genome editing technology on the enhancement of crop plants for abiotic stress tolerance. The current progress and challenges of CRISPR-Cas mediated genome editing approach are discussed in relation to breeding climate-smart wheat for ensuring global food and nutritional security (Afroz et al., 2023).

Currently, CRISPR/Cas-based genome engineering has been successfully used to understand the genetic mechanism underlying tolerance against multiple abiotic stresses, including drought, salinity, heat, and nutritional values in various crop plants (Shi et al., 2017; Li et al., 2019). In this review, we summarize most potential applications of the CRISPR/Cas9-mediated genome editing approach in crop plants aimed at managing abiotic stresses such as drought, salinity, and heat. We also discuss the future opportunities for the applications of CRISPR/Cas-based systems for developing stress-tolerant crop varieties.

## 2 Mechanistic overview of CRISPR/Cas9-based genome editing technology

CRISPR/Cas system is based on an adaptive immune system discovered in bacterial and archaeal genomes to protect against the invasion of foreign plasmids or viral DNA (Marraffini and Sontheimer, 2010). CRISPR/Cas9 is a two-component system comprising CRISPR-associated protein 9 (Cas9) and a single guide RNA (sgRNA) (Cong et al., 2013). The sgRNA is a synthetic combination of two different RNAs necessary for CRISPR activity, the protospacer-matching CRISPR RNA (crRNA) and the transactivating crRNA. The 20 nucleotides at the 5’ end of a sgRNA as a component of the sgRNA/Cas9 complex, bind to the target genome site. This specific target site must be located immediately upstream of the protospacer adjacent motif (PAM; NGG for SpCas9 from *Streptococcus pyogenes*), i.e., a short (typically 2-5 base-pair length) conserved DNA sequence downstream to the cleavage site and its size alters based on the bacterial species. The SpCas9 protein is a large (1368 amino acids) multi-domain DNA endonuclease accountable for the cleavage of target DNA in the genome that produces a blunt-ended double-strand break (DSB). Finally, the DSB is repaired by the host cellular machinery (Mei et al., 2016).

**Figure 1:**
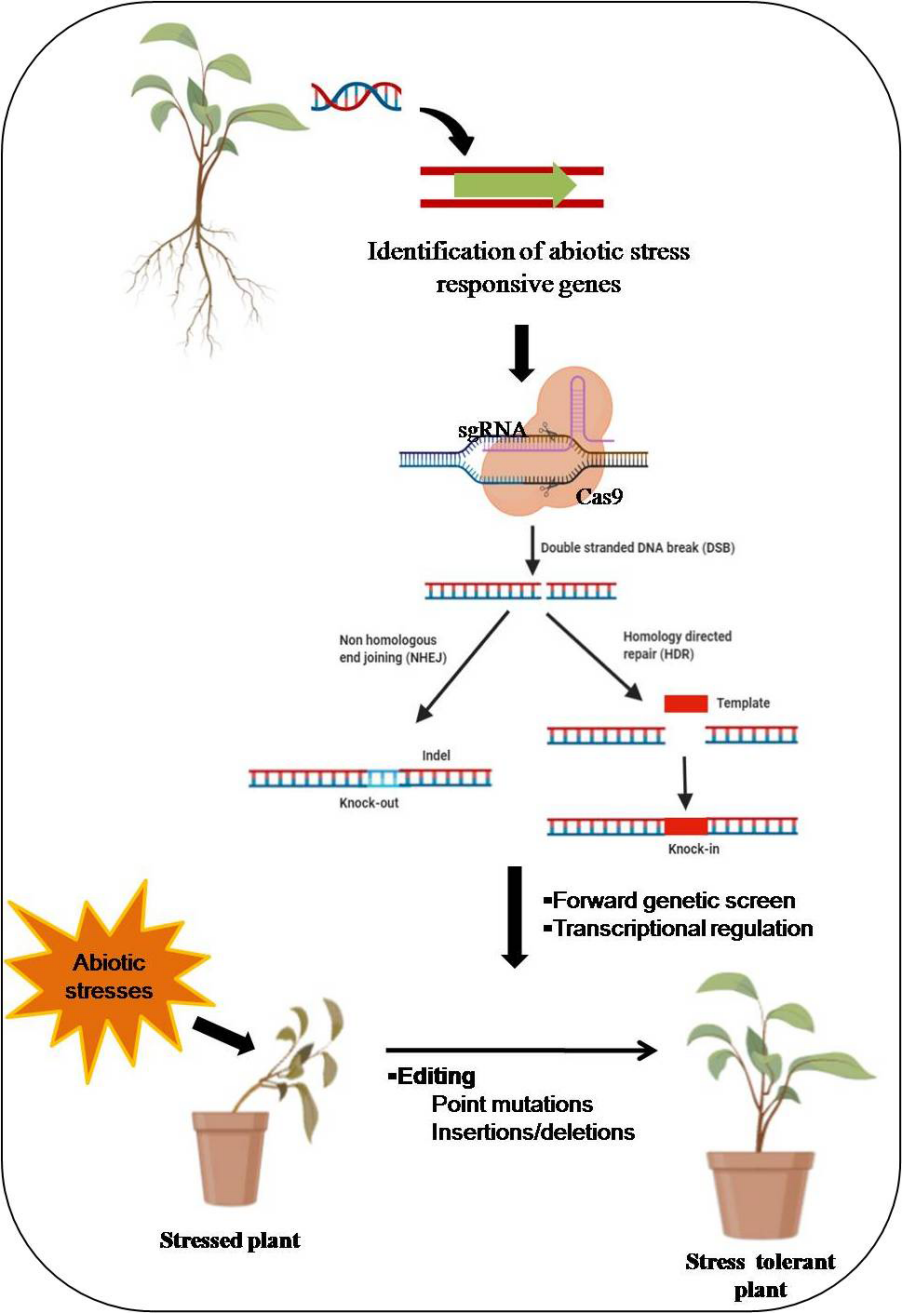
Schematic display of mechanistic insights of CRISPR/Cas9-based genome editing in plants. The Cas9 protein is guided by a desired single guide RNA (sgRNA) and creates a double-strand break (DSB). Subsequently, DNA repair occurs through non-homologous end-joining (NHEJ) or homology-directed repair (HDR) pathways. Figure created with BioRender.com (https://app.biorender.com/biorender-templates)-accessed on 25 May 2022.

The DSBs formed by Cas-9 protein are repaired by two pathways, i.e., homology-directed repair (HDR) and non-homologous end joining (NHEJ) mechanisms (Liu et al., 2018). Homology-directed repair is exceptionally accurate, and it employs a homologous DNA template. HDR is mainly active in the cell cycle’s late S and G2 phases and needs a large amount of donor DNA templates containing a target DNA sequence. It implements the specific gene insertion or replacement by adding a donor DNA template with sequence homology at the predicted DSB site (Liu et al., 2018; Yang et al., 2020). Non-homologous end-joining expedites the DSB’s repairs by joining DNA fragments using an enzymatic procedure without exogenous homologous DNA. **Figure 1** shows the mechanistic scheme of CRISPR/Cas9-based genome editing in plants. A homology-directed repair proceeds through homologous recombination (HR), is usually the ideal gene-editing tool. It can enable error-free editing by integrating a sequence provided by the donor template. It is one of the most common genome editing strategies for biological applications such as knock-in or precise mutagenesis. Conversely, the low frequency of native HR remains the major hurdle to achieve efficient genome editing in plants. HDR-mediated Cas9 can introduce resistance (R) genes to improve stress response in plants. In the HDR repair mechanism, homologous donor DNA is required to repair damaged DNA, and it is more precise than NHEJ, an error-prone DNA repair mechanism. NHEJ introduces indels, and HDR can be used to introduce specific point mutations or insertion of desired sequences *via* homologous recombination (Jain, 2015).

Recent years have witnessed various technological advances that have eased the development and analysis of CRISPR-edited crop plants. A major technical bottleneck to CRISPR application in plants is the low innate HDR efficiency, which obstructs numerous proposed applications, e.g., gene replacement and large chromosomal deletions. Various technologies have been developed to enhance HDR but it has limitations. Ali et al., 2020 generated a fusion between that Cas9 and *VirD2* that produces targeted and specific DNA DSBs and *VirD2* relaxase which allows the repair template in close proximity to the DSBs to facilitate HDR (Ali et al., 2020).Editing of multiple alleles of the *ACETOLACTATE SYNTHASE* (ALS) gene induced through nuclease CRISPR/Cas9 in sugarcane led to increased herbicide tolerance (Oz et al., 2021). This specific replacement of the target ALS gene in sugarcane *via* template-mediated and HDR of DSB provides herbicide resistance to these CRISPR-edited plants. The pyramiding/stacking of multiple genes involved in a stress response pathway or regulatory network can be done *via* HDR-mediated gene targeting. Higher HDR efficiency was achieved when tandemly repeated sequences existed near DSBs and then developed a tandem repeat-HDR strategy (TR-HDR) for gene targeting. This TR-HDR successfully introduced in-locus tags, with editing efficiencies ranging from 3.4 to 11.4% in rice (Ali et al., 2020).

Additionally, the Cas9-VirD2 fusion protein improved the efficiency of HDR repair up to 4-fold than Cas9 alone and allowed precise alteration of the *OsALS* allele, the *OsCCD7* gene, and to make an in-frame epitope tag fusion at *OsHDT* to develop herbicide tolerance in rice(Ali et al., 2020).

## 3 Novel technological approaches and strategic suggestions for genome editing

Novel gene editing approaches evolved from the CRISPRs-Cas9, base editing (BE), and prime editing (PE) technologies open new avenues for the functional analysis of genes. For example, the editing efficiency of Cas9 could be improved through competent screening of targeted characteristics, investigating genetic material through gene knock-out, and using an ultimate genetic transformation procedure. In this section, we present latest breakthroughs achieved in the field of genome editing of crop plants.

### 3.1 Base editing and prime editing

Many important crop traits can be improved with a single base change in the genes and do not require DSBs and donor DNA templates for HDR. Single-base editing in such situations cannot be accomplished with the knock-in/out approach based on regular CRISPR-Cas system. Studies on agronomic traits suggest that numerous such traits are resolute by changes in the single bases of genes (Li et al., 2017). The CRISPR/Cas9 system has limitations and cannot be used to carry out gene base conversion. Therefore, it is the most suitable for knock-out or knock-in genes in the genome. Considering these restrictions, it is vital to find an accurate and robust method for editing the crop plant genomes.

A novel editing strategy that serves this purpose is base editing (Veillet et al., 2019), which accurately brings nucleotide changes in the absence of DSBs (Yin et al., 2017; Davies, 2019). The base editing pipeline still relies on the gRNA-guided target finding in the genome, however, involving the inactive CRISPR–Cas9 nuclease (which cannot make DSBs) fused to the deaminase enzyme (cytosine or adenosine) component that manipulates the nucleotide conversion.

A newly evolved cytidine base-editing tool proposes a valuable substitute (Komor et al., 2016). In wheat, acetolactate synthase *(ALS)* is an ideal herbicide tolerance target gene for base editing that can contain point mutations conferring adequate herbicide tolerance with a minor consequence to plant productivity(Yu and Powles, 2014). This is why mutations in ALS genes have been obtained through cytidine base editing in some diploid plant species, including Imidazolinone tolerance in rice (Shimatani et al., 2017)and tribenuron tolerance in *Arabidopsis* (Chen et al., 2010).

Compared to HDR-dependent CRISPR/Cas9, prime editing (PE) is homology-directed repair (HDR)-independent gene-editing technology that utilizes nCas9 attached to an engineered reverse transcriptase. At the same time, template RNA is linked to sgRNA to custom prime editing guidance RNA (pegRNA), which both stipulates the target site and encodes the anticipated editing sequence. Anzalone et al. (2019) reported that pairs of pegRNAare capable of exactly deleting 710 bps or accurately replacing a sequence of 108 bps. To date, prime editing has been applied to corn, rice, wheat, and tomato (Jiang et al., 2020; Lin et al., 2020; Lu et al., 2021a).

### 3.2 Transgene-free genome editing

Genome-editing has been extensively used across plant species to study and incorporate functional mutations for crop improvement. Conversely, the integration of transgene in the genome of plants is likely to attract considerable public attention and fall under the regulatory frameworks that control use of genetically modified organisms (GMOs)(Gu et al., 2021). Conventional genome engineering methods need the transfer and combination of DNA cassettes to encode modified parts into the host genome. DNA fragments are generally degenerated but generate detrimentally effects (Kim et al., 2014). Transgene free genome editing is becoming a fast-expanding movement in biological sciences because of its advantages. This technique opened the roads to targeted genome alterations without conflict with the genome and created opportunities to deliver non-genetically modified organisms (Zhang et al., 2016a; Rather et al., 2022). However, transgene free genome editing comes across the same fundamental issues as transformation approaches. Regardless of the broader arsenal of transformation techniques, the RNA and protein delivery methodologies are less developed for plant cells than for animals. Thus, only the biolistic method and protoplast transfection could be used to generate transgene-free genome-edited plants (Tsanova et al., 2021). Protoplasts were the primary tissue successfully targeted for DNA-free genome editing through polyethylene glycol (PEG) mediated fusion. Hence, ribonucleoprotein (RNP) complex or mRNA mix with PEG and combine with the protoplast.

Trans-gene free genome editing was studied by transfecting guide RNA and Cas9 protein into protoplasts of tobacco, Arabidopsis, rice, and lettuce and achieved targeted mutagenesis in regenerated plants at frequencies of up to 46% (Woo et al., 2015). Furthermore, to achieve DNA-free genome-edited plants, the wheat embryo has been used for particle bombardment using CRISPR/Cas9 RNAs (Zhang et al., 2016a).This study achieved highly efficient and precise DNA-free genome editing by a transient expression that produced homozygous mutant plants in the T0 generation. In addition, a recently published report by (Rather et al., 2022)revealed an efficient method of DNA-free genome editing in potato (*Solanum tuberosum*) protoplast using circular and linearized plasmid DNA fragments that showed high expression of the transgene and upto 95% gene editing events in protoplast derived potato calli (Rather et al., 2022).

### 3.3 Multiplexed CRISPR technologies for gene editing

Multiplex genome-editing technologies are versatile and powerful tools for precisely modifying numerous specific loci in the genome. In this technique, various gRNA and Cas9 enzymes are expressed at once (Wolter and Puchta, 2017), thus facilitating potent bioengineering applications and greatly improving genome-editing efficiencies (Jacobs et al., 2017). Furthermore, these approaches have significantly enhanced the likelihood of obtaining desired alterations at multiple nucleotide levels in the target genome. With numerous sgRNA targets, several genes can be modified concurrently in any crop plant. Using this technique, various traits could be introduced into new plant varieties. Furthermore, numerous individuals from many families could be targeted by combining multiple sgRNAs into a plasmid vector (Komor et al., 2017; Meng et al., 2017). The principal advantage of CRISPR is its ability to allow multiplex genome editing *via* editing multiple sgRNA targets in the genome. In plants, the application of multiplex genome editing has been demonstrated for improving traits like herbicide tolerance. Nevertheless, the applications are now being extended to cover multiple aspects of plant improvement including metabolic engineering and hormone biosynthesis and perception, and molecular farming capturing >100 concurrent targeting events (Armario Najera et al., 2019)Therefore, multiple genome editing technologies will accelerate creation and utilization of novel genetic variations for faster breeding of new crop varieties for unpredictable and changing climates.

### 3.4 Production of mutant libraries

The greater efficiency of CRISPR-mediated mutagenesis of crop plants allows the improvement of the high-throughput mutagenesis approach. The common use of CRISPR/Cas9 is important for designing mutant libraries to learn the genetic means behind crop improvement. In traditional mutagenesis process, a plant receives many mutations in the genomic background in addition to the targeted site. This leads to a weak association between the genotype and phenotype and further lowers the success of gene identification and cloning (Meng et al., 2017). The preparation of mutant libraries is an efficient and promising tool (Lu et al., 2017). CRISPR-Cas based mutant production has an advantage over chemical-based mutation for being more site-specific in the genome and having an strong association to the phenotype of interest. Genome-scale mutant library *via* CRISPR/Cas9 are now available for *Arabidopsis* (Peterson et al., 2016), tomato (Jacobs et al., 2017) and in rice (Meng et al., 2017). The mutant libraries are prepared by changing the 18-20 bp target binding order in the sgRNA target. Tomato transformation was carried out by transforming pooled CRISPR libraries to generate a group of mutant lines with the least transformation attempts and in less time (Jacobs et al., 2017). In this study, a single transformation attempt was performed using the CRISPR library that targeted immunity-related leucine-rich repeat subfamily XII genes resulting in inherited mutation retrieving in 15 of the 54 targeted genes.

Further, to improve productivity, they constructed a second library containing three sgRNAs per construct to target 18 genes, resulting in mutagenesis in 15 of 18 targeted genes (Jacobs et al., 2017). For the rice plant transformation, mutant libraries were generated with loss-of-function mutation (Meng et al., 2017). These plants showed phenotypic changes like lethality and sterility during their cultivation in the field. Overall, mutant libraries are a powerful tool for improving genome-editing techniques for abiotic stress management in crops. By screening mutant libraries for stress tolerance and identifying targets for genome editing, researchers can develop more effective strategies for improving stress tolerance in crops.

## 4 Impact of CRISPR/Cas9-based genome editing on plant productivity and stress tolerance

Abiotic stress negatively affects plant growth and crop production (Jha et al., 2020)by impairing with diverse biochemical, morphological, and physiological parameters crucial for plant growth. So far, CRISPR-Cas mediated gennome editing is broadly used and adopted in almost 20 agronomically essential crops. The CRISPR/Cas9editing technology has the potential to significantly impact plant productivity and abiotic stress tolerance. By using CRISPR/Cas9, scientists can target specific genes involved in stress response pathways and modify them to enhance the plant’s ability to withstand adverse environmental conditions. Abiotic stress tolerance is mostly governed by QTLs with several genes (Jha et al., 2020). Since many of the time it is harder to pinpoint the causal gene, plant breeders prefer to use QTL or relatively a broad chromosomal region to improve a variety. In this situation breeding pipeline incorporate many undesired traits that may have negative impact on the crop performance. The CRISPR/Cas9-based genome editing is more precise to genomic targets and less likely to change the genomic background of a variety. With the improved bioinformatics tools and functional studies, the negative regulators of abiotic stress can be targeted for editing by CRISPR/Cas9. Moreover, the non-desired gene in QTL can be edited for loss of function without their physical separation from the desired gene to prevent the off-type phenotype. In situations, where the abiotic tolerance in a variety is governed by gene with variation in few SNPs, the advanced CRISPR tools can be used to alter SNPs to produce the functional protein for trait augmentation in the variety circumventing the need for time consuming plant breeding procedures involving crossing and selections.

**Figure 2:**
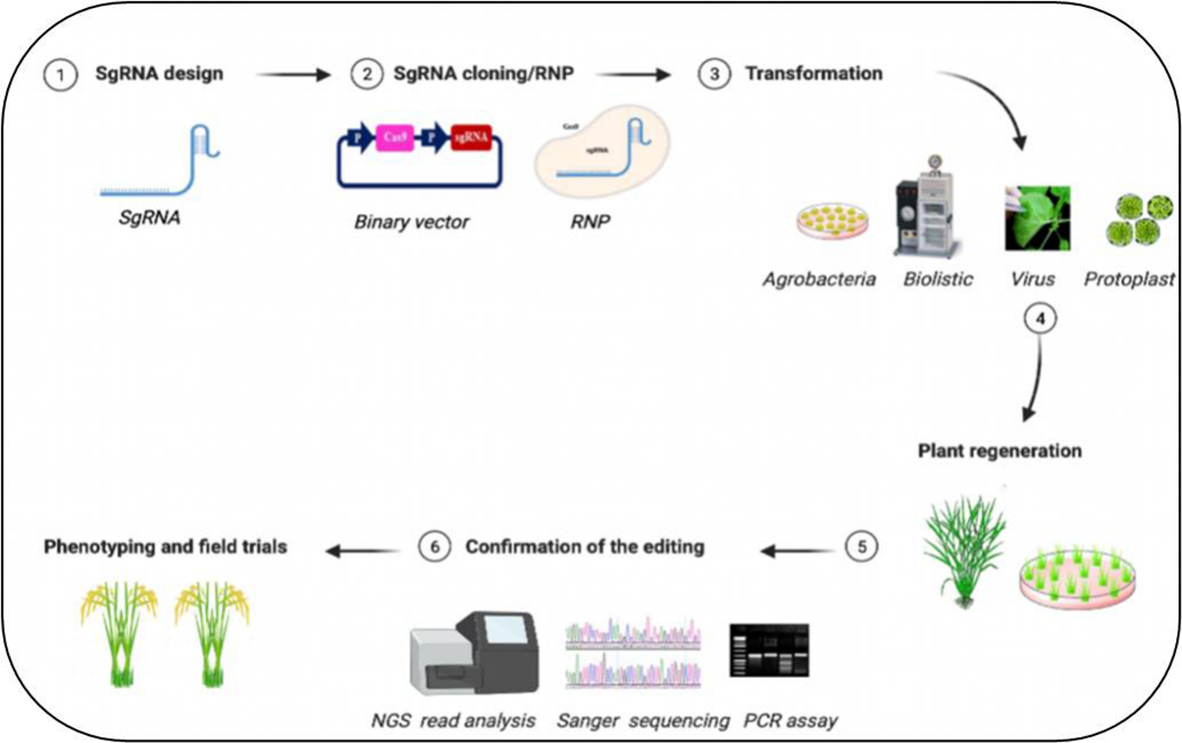
Simplified workflow for CRISPR/Cas9-mediated plant genome editing. The generation of edited plants with the desired phenotype starts with the design of (1) guide RNA (gRNA) for a specific target sequence and (2) cloning of the sequence to express the sgRNA into a binary vector containing the Cas DNA sequence or forming ribonucleic protein complex (RNP). Then the (3) delivery of CRISPR/Cas materials into the plant tissues through various methods, (4) regeneration of the transgenic plants followed by (5) assays to confirm the editing events with (6) improved trait of crop plants. Figure created with BioRender.com (https://app.biorender.com/biorender-templates)—accessed on 25 May 2022.

CRISPR/Cas9-based editing of abiotic stresses, including drought, salt, heat, cold, and heavy metal stress-responsive genes and their negative regulators, and mode of plant transformation for development of stress tolerance in plants have been listed in **Tables 1**–**3**. A simplified workflow for CRISPR/Cas9-mediated genome editing in plants has been displayed in **Figure 2**.

**Table 1:** Application of the CRISPR-based genome editing approach in plants for improvement of drought and salinity stress tolerance.

Stress Tolerance	Plant species	Target Gene	Gene ID	Method of Delivery	Reference	
Drought tolerance	*Arabidopsis thaliana*	*AtOST2*	NM_001335616	Agrobacterium- Mediated	(Osakabe et al., 2016)	
Drought Tolerance	*A. thaliana*	*AtAREB1*	AT1G45249.3	Agrobactrium -mediated	(Roca Paixão et al., 2019)	
Drought Tolerance	*A. thaliana*	*AtAVP1*	NM_101437	Agrobacterium- Mediated	(Park et al., 2017)	
Drought Tolerance	*A. thaliana*	*AtmiR169a*	–	Agrobacterium- Mediated	(Zhao et al., 2016)	
Drought tolerance	*Brassica napus*	*BnaA6.RGA*	LOC106445425	Agrobacterium- Mediated	(Wu et al., 2020)	
Drought Tolerance	*Cicer arietinum*	*At4CL*, *AtRVE7*	LOC101502718, LOC101509066	PEG-mediated	(Badhan et al., 2021)	
Drought tolerance	*Glycine max*	*GmMYB118*	GLYMA_17G094400	Agrobacterium- Mediated	(Du et al., 2018)	
Drought tolerance	*Oryza sativa*	*OsERA1*	LOC_Os01g53600	Agrobacterium- Mediated	(Ogata et al., 2020)	
Drought tolerance	*O. sativa*	*OsSAPK2*	LOC_Os07g42940	Agrobacterium- mediated	(Lou et al., 2017)	
Drought tolerance	*O. sativa*	*OsSRL1, OsSRL2*	LOC_Os01g54390	Agrobacterium- mediated	(Liao et al., 2019)	
Drought Tolerance	*O. sativa*	*OsDST*	LOC_Os03g57240	Agrobacterium- Mediated	(Santosh Kumar et al., 2020)	
Drought Tolerance	*O. sativa*	*OsNAC14*	Os01g0675800	Agrobacterium- Mediated	(Shim et al., 2018)	
Drought Tolerance	*O. sativa*	*OsPUB67*	*NP_001065331.1*	Agrobacterium- Mediated	(Qin et al., 2020)	
Drought tolerance	*Solanum* *lycopersicum*	*SlNPR1*	KX198701	Agrobacterium- Mediated	(Li et al., 2019)	
Drought Tolerance	*S.* *lycopersicum*	*SlMAPK3*	AY261514	Agrobacterium- Mediated	(Wang et al., 2018)	
Drought Tolerance	*S.* *lycopersicum*	*SlLBD40*	Solyc02g085910	Agrobacterium- Mediated	(Liu et al., 2020)	
Drought Tolerance	*S.* *lycopersicum*	*SlARF4*	Solyc11g069190	Agrobacterium- Mediated	(Chen et al., 2021)	
Drought tolerance	*Triticum* *Aestivum*	*TaDREB2, TaDREB3, TaERF3*	DQ353852.1 EF570122.1	PEG-mediated	(Kim et al., 2018)	
Drought Tolerance	*T.* *Aestivum*	*TaDREB2, TaERF3*	DQ353852.1, EF570122.1	Agrobacterium- Mediated	(Kim et al., 2018)	
Drought tolerance	*Zea mays*	*ZmARGOS8*	GQ184457	Agrobacterium- mediated	(Shi et al., 2017)	
Salt tolerance	*A.* *Thaliana*	*AtWRKY*, *AtWRKY4*	–	Agrobacterium- Mediated	(Li et al., 2021b)	
Salt tolerance	*A.* *Thaliana*	*AtACQOS*	*AT5G46510*	Agrobacterium- Mediated	(Kim et al., 2021)	
Salt tolerance	*Glycine max*	*GmDrb2a, GmDrb2b*	NM_001254313	Agrobacterium- Mediated	(Curtin et al., 2018)	
Salt tolerance	*G. max*	*GmAITR*	XM_003549793	Agrobacterium- Mediated	(Wang et al., 2021b)	
Salt tolerance	*Medicago* *Truncatula*	*MtHEN1*	Medtr4g094545	Agrobacterium- Mediated	(Curtin et al., 2018)	
Salt tolerance	*O. sativa*	*OsDST*	LOC_Os03g57240	Agrobacterium- Mediated	(Santosh Kumar et al., 2020)	
Salt tolerance	*O. sativa*	*OsRAV2*	LOC_Os01g04800	Agrobacterium- Mediated	(Duan et al., 2016)	
Salt tolerance	*O. sativa*	*OsRR22*	KF892986	Agrobacterium- Mediated	(Zhang et al., 2019)
Salt tolerance	*O. sativa*	*OsNAC45*	KT957809	Agrobacterium- Mediated	(Zhang et al., 2020)
Salt tolerance	*O. sativa*	*OsBBS1*	LOC_Os03g24930	Agrobacterium- Mediated	(Zeng et al., 2018)
Salt tolerance	*O. sativa*	*OsAGO2*	LOC4336991	Agrobacterium- Mediated	(Yin et al., 2020)
Salt tolerance	*O. sativa*	*OsVDE*	*LOC_Os04g31040*	Agrobacterium- Mediated	(Wang et al., 2003)
Salt tolerance	*O. sativa*	*OsNAC041*	LOC_Os03g013300	Agrobacterium- Mediated	(Bo et al., 2019)
Salt tolerance	*O. sativa*	*OsSAPK2*	LOC_Os07g42940	Agrobacterium- Mediated	(Lou et al., 2017)
Salt tolerance	*O. sativa*	*OsPQT3*	LOC_Os10g29560.1	Agrobacterium- Mediated	(Alfatih et al., 2020)
Salt tolerance	*O. sativa*	*OsPIL14*	LOC_Os07g05010	Agrobacterium- Mediated	(Mo et al., 2020)
Salt tolerance	*O. sativa*	*OsBGE3*	LOC_Os01g48800	Agrobacterium- Mediated	(Yin et al., 2017)
Salt tolerance	*O. sativa*	*OsSPL10*	LOC_Os06g44860	Agrobacterium- Mediated	(Lan et al., 2019)
Salt tolerance	*O. sativa*	*OsDOF15*	LOC_Os03g55610	Agrobacterium- Mediated	(Qin et al., 2019)
Salt tolerance	*O. sativa*	*OsFLN2*	AP014960	Agrobacterium- Mediated	(Chen et al., 2020)
Salt tolerance	*S.* *tuberosum*	*StCoilin*	LOC102603469	PEG-mediated	(Makhotenko et al., 2019)
Salt tolerance	*S.* *lycopersicum*	*SlHyPRP1*	LOC101257680	PEG-mediated	(Tran et al., 2021)
Salt tolerance	*T.* *aestivum*	*TaHAG1*	TraesCS1D02G134200	Agrobacterium- Mediated	(Zheng et al., 2021)

-, ID not found.

**Table 2:** Application of the CRISPR-based genome editing approach in plants for improvement of heat and cold stress tolerance.

Stress Tolerance	Plant species	Target Gene	Gene ID	Method of Delivery	Reference
Heat tolerance	*Gossypium* *hirsutum*	*GhPGF, GhCLA1*	–	Agrobacterium- Mediated	(Li et al., 2021a)
Heat tolerance	*Lactuca sativa*	*LsNCED4*	LOC111879595	Agrobacterium- Mediated	(Bertier et al., 2018)
Heat tolerance	*O. sativa*	*OsPDS*	LOC_Os03g08570	Gene gun	(Nandy et al., 2019)
Heat tolerance	*O. sativa*	*OsHSA1*	XM_026023654	Agrobacterium- Mediated	(Qiu et al., 2018)
Heat tolerance	*O. sativa*	*OsNAC006*	–	PEG-mediated	(Wang et al., 2020)
Heat tolerance	*O. sativa*	*OsPYL1/4/6*	–	Agrobacterium Mediated	(Miao et al., 2018)
Heat tolerance	*S.* *lycopersicum*	*SIAGL6*	Solyc01g093960	Agrobacterium- Mediated	(Klap et al., 2017)
Heat tolerance	*S. lycopersicum*	*SlCPK28*	Solyc02g083850	Agrobacterium- Mediated	(Hu et al., 2021)
Heat tolerance	*S. lycopersicum*	*SlMAPK3*	NM_001247431.2	Agrobacterium- Mediated	(Yu et al., 2019)
Heat tolerance	*S. lycopersicum*	*SlBZR1*	Solyc04g079980	Agrobacterium- Mediated	(Yin et al., 2018)
Heat tolerance	*Z. mays*	*ZmTMS5 gene*	–	particle bombardment	(Li et al., 2017)
Cold tolerance	*A. thaliana*	*AtCBF1, AtCBF2*	AT4G25490, AT4G25470	Agrobacterium- Mediated	(Chen et al., 2010)
Cold tolerance	*O. sativa*	*OsAnn3*	LOC_Os07g46550	Agrobacterium- Mediated	(Shen et al., 2017)
Cold tolerance	*O. sativa*	*OsPIN5b, GS3, OsMYB30*	Os08g0529000, Os03g0407400, Os02g0624300	Agrobacterium- Mediated	(Zeng et al., 2020)
Cold tolerance	*O. sativa*	*OsAnn5*	–	Agrobacterium- Mediated	(Shen et al., 2017)
Cold tolerance	*O. sativa*	*OsPRP1*	AB055842	Agrobacterium- Mediated	(Nawaz et al., 2019)
Cold tolerance	*S. lycopersicum*	*SlCBF1*	–	Agrobacterium- Mediated	(Li et al., 2018a)

-, ID not found.

**Table 3:** Application of the CRISPR-based genome editing approach in plants for improvement of metals and herbicide stress tolerance.

Stress Tolerance	Plant species	Target Gene	Gene ID	Method of Delivery	Reference
Metal stress tolerance	*A. thaliana*	*Atoxp1*	At5G37830	Agrobacterium- mediated	(Baeg et al., 2021)
Metal stress tolerance	*O. sativa*	*OsARM1*	Os05g37060	Agrobacterium- mediated	(Wang et al., 2017a)
Metal stress tolerance	*O. sativa*	*OsNramp5*	Os07g0257200	Agrobacterium- mediated	(Tang et al., 2017b)
Metal stress tolerance	*O. sativa*	*OsLCT1*	AB905363	Agrobacterium- mediated	(Lu et al., 2017)
Metal stress tolerance	*O. sativa*	*OsHAK1*	Os04g32920	Agrobacterium- Mediated	(Nieves-Cordones et al., 2017)
Metal stress tolerance	*O. sativa*	*OsPRX2*	Os02g053770	Agrobacterium- Mediated	(Mao et al., 2019)
Metal stress tolerance	*O. sativa*	OsATX1	–	Agrobacterium- Mediated	(Zhang et al., 2021b)
Herbicide resistance	*B. napus*	*BnALS*	LOC106353716	Agrobacterium- Mediated	(Wu et al., 2020)
Herbicide resistance	*Manihot* *esculenta*	*MeEPSPS*	Manes.05G046900	Agrobacterium- Mediated	(Hummel et al., 2018)
Herbicide resistance	*O. sativa*	*OsALS*	LOC4329938	Agrobacterium- Mediated	(Zhang et al., 2021a)
Herbicide resistance	*O. sativa*	*OsALS*	MN268687	Agrobacterium- Mediated	(Wang et al., 2021a)
Herbicide resistance	*O. sativa*	*OsTB1*	AF322143	Agrobacterium- Mediated	(Butt et al., 2020)
Herbicide resistance	*O. sativa*	*OsPUT1/2/3*	Os02g0700500, Os12g0580400,Os03g0576900	Agrobacterium- Mediated	(Lyu et al., 2022)
Herbicide resistance	*O. sativa*	*OsACC*	LOC_Os05g22940	Agrobacterium- Mediated	(Liu et al., 2018)
Herbicide resistance	*O. sativa*	*OsEPSPS*	AF413081	PEG-mediated	(Li et al., 2016)
Herbicide resistance	*O. sativa*	*OsEPSPS*	AF413081	biolistic gene transfer	(Li et al., 2016)
Herbicide resistance	*O. sativa*	*Os*ALS*-1,Os*ALS*-2, Os*ALS*-3,Os*ALS*-4*	–	Agrobacterium- Mediated	(Endo et al., 2016)
Herbicide resistance	*Saccharum officinarum*	*SoALS*	MZ268741	biolistic gene transfer	(Oz et al., 2021)
Herbicide resistance	*S. lycopersicum*	*SlEPSPS*	Solyc01g091190	Agrobacterium- Mediated	(Yang et al., 2022)
Herbicide resistance	*S. lycopersicum*	*SlALS1, SlALS2*,	Solyc06g059880, Solyc03g044330	Agrobacterium- Mediated	(Yang et al., 2022)
Herbicide resistance	*S. lycopersicum*	*Slpds1*	Solyc03g123760	Agrobacterium- Mediated	(Yang et al., 2022)
Herbicide resistance	*T. aestivum*	*TaALS*	TraesCS6A02G288000	Biolistic-mediated	(Zhang et al., 2019)
Herbicide resistance	*Z. mays*	*ZmALS1*, *ZmALS2*	LOC100381801, LOC100274341	Agrobacterium- Mediated	(Svitashev et al., 2015)
Herbicide resistance	*Z. mays*	*MS26*	LOC100191749	Biolistic-mediated	(Svitashev et al., 2015)

-, ID not found.

### 4.1 Drought stress tolerance

Overexpression of several drought-responsive genes and transcription factors increases the accumulation of signaling molecules and metabolic compounds and enhances drought tolerance in plants (Fang and Xiong, 2015; Kumar et al., 2019; Santosh Kumar et al., 2020). The expressions of drought-sensitive (S) genes enhance susceptibility in plants to drought through hormonal disproportion, declined antioxidant activities, and increasing reactive oxygen species (ROS) production. Over-expression of *AREB1* has shown improved tolerance to drought stress, whereas the *AREB1* knock-out mutant had higher sensitivity to drought stress (Singh and Laxmi, 2015). CRISPR/Cas9 targeted mutagenesis of *SlLBD40*, a lateral organ boundaries domain transcription factor that enhances drought tolerance in tomatoes compared with overexpressing transgenic and WT tomato plants; knockout of *SlLBD40* by CRISPR/Cas9 enhanced the drought tolerance of tomato (Liu et al., 2020). CRISPR/Cas9-edited tomato (*Solanum lycopersicum*) mutant plants knock-out for *SlMAPK3* gene had enhanced drought stress response (Wang et al., 2017b). Under drought, these mutant plants exhibited severe wilting symptoms, elevated levels of H_2_O_2_, reduced antioxidants, and increased membrane damage. These results substantiate that *SlMAPK3*is implicated in drought stress response in tomato plants by protecting the cell membrane. Knockout of the tomato *Auxin Response Factor* (*SlARF4)* gene improves tomato resistance to water deficit (Chen et al., 2021). Arabidopsis *histone acetyltransferase 1 (AtHAT1)* promotes gene expression activation by switching chromatin to a relaxed state. Improved drought stress tolerance was observed in Arabidopsis by CRISPR/dCas9 fusion with a *Histone Acetyl Transferase* (*AtHAT*) gene (Roca Paixão et al., 2019). CRISPR/Cas9-based editing of *pathogenesis-related 1 (NPR1*) gene in tomato exhibited drought response (Li et al., 2019) by enhancing stomatal aperture, malondialdehyde (MDA) level, H_2_O_2_ content and ion leakage. However, the antioxidant activity level declined more than in wild type plants. The *SlNPR1* plays a significant role in directing responses against drought stress in tomato and other crop plants. Multiple *SlNPR1* variants can be developed through genome editing to enhance drought tolerance in a wide range (Li et al., 2019). Drought-induced SINA protein 1 *(OsDIS1*), drought and salt-tolerant protein 1 *(OsDST*), and ring finger protein1 (*OsSRFP1*) genes are negative regulators of drought tolerance. Silencing these drought-responsive genes improved levels of antioxidant enzymes, decreased concentrations of H_2_O_2_, and increased tolerance to drought stress in rice plants (Fang and Xiong, 2015; Santosh Kumar et al., 2020). *Enhanced Response 1 (ERA1*) protein gene regulates plants’ ABA signaling and dehydration responses. In rice, editing of the *OsERA1*gene enhanced tolerance to drought stress, with the mutant plants showing increased sensitivity to ABA and stomatal closure under drought conditions (Ogata et al., 2020). *OsSAPK2* is also involved in ABA-mediated stress tolerance in rice and its participation was confirmed by developing mutants using CRISPR/Cas9 with loss of function mutation. The mutants exhibited more drought sensitivity than WT plants (Lou et al., 2017).

Transcription factor OsWRKY5 inhibits the ability to withstand drought. OsWRKY5 was mostly expressed in growing leaves throughout the seedling and heading phases, and drought stress decreased its expression. Plant growth under water shortage was used as a measure of the increased drought tolerance imparted by the genome-edited loss-of-function alleles *oswrky5-2* and *oswrky5-3* (Lim et al., 2022). In contrast, greater susceptibility was observed under the same circumstances when *OsWRKY5* was overexpressed in the activation-tagged line *oswrky5-D*. *OsWRKY5* activity was lost, increasing sensitivity to ABA and encouraging ABA-dependent stomatal closure. *OsWRKY5* genome editing increases grain production under drought stress.

The CRISPR Cas9-induced mutations in the gene encoding OPEN STOMATA 2 (*AtOST2*) in Arabidopsis mutants facilitated an enhanced stomatal response than WT (Osakabe et al., 2016).

Interestingly, the *AtOST2* mutants had a high degree of stomatal closure (Osakabe et al., 2016). In rice, *OsSRL1* and *OsSRL2* gene encodes leaf tissue phenotype. The genome-modified lines having homozygous *SRL1* and *SRL2* mutants were found retardation in various characteristics such as the stomata number, stomatal conductance, transpiration rate, chlorophyll content, vascular bundles and other agronomic traits in comparison to wild-type one (Liao et al., 2019). Drought tolerance can be obtained through CRISPR/Cas9-based genome editing by targeting negative regulators or drought-sensitive genes. CRISPR/Cas9-based genome editing in *Zea mays* was carried out to enhance the expression level of the *ARGOS8* gene, which negatively regulates ethylene response, for the development of drought tolerance. Such mutant plants showed improved grain yields in the field under drought-stress conditions (Hirai et al., 2007). WRKY transcription factors regulate the plant’s growth and development and involve biotic and abiotic stresses. In plants, *WRKY3* and *WRKY4* genes play an important role in regulating defense response to drought stress (Li et al., 2021b).

### 4.2 Salinity stress tolerance

By the year 2050, more than 50% of agricultural lands may get critically salinized (Wang et al., 2003; Kaashyap et al., 2017; Jha et al., 2019). In plants, salt stress causes various physiological and morphological changes because of alterations in the expression of genes and signaling pathways (Prusty et al., 2018). The key detrimental effects of salinity stress are necrosis, untimely death of old leaves, and harsh interruption of ions in cells (Julkowska and Testerink, 2015). Several genes have been identified and characterized through CRISPR/Cas-based genome editing to improve plant salt tolerance. Knockout of *AtWRKY3* and *AtWRKY4* genes in *A. thaliana* plants using CRISPR/Cas9 exhibited significant up-regulation of genes under salt and methyl jasmonate stresses. Such double mutant plants showed sensitivity features to salinity and methyl jasmonate, such as elevation in ion leakage and reduction in antioxidant activities, including peroxidase (POD), catalase (CAT), and superoxide dismutase (SOD)(Chen et al., 2021) (**Table 1**). Also, the importance of the *Acquired Osmotolerance* (*AtACQOS*) gene provided tolerance against salt stress in *Arabidopsis* is characterized by CRISPR-generated mutants (Kim et al., 2021). CRISPR/Cas9-based knock-out mutants of abscisic acid (ABA)-induced transcription repressors (AITRs) genes conferred salt stress tolerance in soybean (*Glycine max*) plant (Wang et al., 2021b).These mutant plants showed increased ABA sensitivity and produced longer roots and shoots than WT plants. Similarly, knock-out mutants of *GmDrb2a* and *GmDrb2b* genes showed enhanced salinity stress tolerance in *G. max* (Curtin et al., 2018). CRISPR/Cas9-mediated editing of *OsRAV2* gene expression was induced by the regulatory function of the GT-1 element in rice and showed tolerance to salt stress (Duan et al., 2016). CRISPR mutants with loss of function of *SnRK2* and osmotic stress/ABA-activated protein kinases *SAPK-1* and*SAPK-2* genes showed resistance to salt stress in rice (Lou et al., 2017). In several other studies, the development of CRISPR-mutants in rice to develop salt stress tolerance plants through knock-out of *OsDST* (Santosh Kumar et al., 2020), *OsNAC45* (Zhang et al., 2020), *AGO2* (*ARGONAUTE2*) (Yin et al., 2020), Rice type-B response regulator (*OsRR22*) (Zhang et al., 2019), and *OsBBS1* (bilateral blade senescenc1) (Zeng et al., 2018) have been carried out. Knock-out mutants of the *TaHAG1* gene of wheat plants generated through CRISPR/Cas9 showed enhanced salt tolerance (Zheng et al., 2021). Alam et al. (2022) generated the gene edited rice mutant plants through targeting *OsbHLH024* gene to study its role under salt stress condition. The deletion of one nucleotide base was observed in the *osbhlh024* mutants its exposure under salt stress resulted in significantly enhance in total chlorophyll content and shoot biomass (Alam et al., 2022).

Violaxanthin de-epoxidase (VDE) plays a critical role in plants’ ABA biosynthesis, growth, and stress responses. In rice, the functional benefits of *OsVDE* in salt tolerance are validated. Gene-editing targeting *OsVDE* loci in overexpressed transgenic rice was found to have a higher ABA level, stomatal closure percentage and survival rate than the wild type under seedling stage salt stress. (Wang et al., 2003). Several plant transcription factor family genes are involved in the salt stress response. NAC transcription factor coding gene, *OsNAC041*, confirmed its importance for germinating seeds under salt stress. *Osnac041* mutant obtained by CRISPR/Cas9 method showed increased salt sensitivity compared to the wild plants (Bo et al., 2019). Transcription factor *OsDOF15* positively regulates primary root elongation by regulating cell proliferation in the root meristem *via* restricting ethylene biosynthesis. Loss-of-function of *OsDOF15* impaired primary root elongation and cell proliferation in the root meristem (Qin et al., 2019). Some instances where editing on the negative regulator for salt stress tolerance are *OsPQT3* and the DELLA protein SLENDER RICE1 (*SLR1*). In rice, *OsPQT3* knockout mutants displayed enhanced oxidative and salt stress resistance to elevated expression of *OsGPX1, OsAPX1* and *OsSOD1* under salt stress (Alfatih et al., 2020), and the loss of function of SLR1 promoted mesocotyl and root growth, specifically in the dark and under salt stress (Mo et al., 2020).

### 4.3 Heat stress tolerance

High temperature or heat stress is one of the major abiotic stresses that become a severe problem in agricultural production in several regions of the world that causes global warming (Tubiello et al., 2007). Plants react to heat stress by activating complex molecular networks, including heat stress-responsive gene expression, signal transduction, and metabolite production (Jha et al., 2017a). With the advancements in functional and structural genomics techniques in plants, various heat stress-associated genes have been identified and characterized to enhance heat tolerance with advanced biotechnological tools. The Heat shock proteins (HSPs) and heat shock transcription factors (HSFs) are crucial gears and function through the heat stress-response signal transduction pathway, which is linked to ROS accumulation (Awasthi et al., 2015). Hence, heat stress tolerance can be improved by enhancing the ability of plants to detoxify ROS components (Parmar et al., 2017). This indicated that enhanced tolerance could improve crop plants’ antioxidant activities. Heat-induced gene expression and metabolite biosynthesis significantly enhanced heat tolerance in plants. Among all the genome-editing approaches, CRISPR/Cas9 is a revolutionary technique for genome editing in a precise manner to learn the molecular pathways associated with heat stress and improve crop heat tolerance (Duan et al., 2016). Tomato (*Solanum lycopersicum*) is considered an ultimate model to test CRISPR/Cas9-based genome editing because it can endure competent transformation to grain quality enhancement (Pan et al., 2016). Currently, CRISPR/Cas9-based genome editing of the heat-sensitive gene, *SlAGAMOUS-LIKE 6* (*SIAGL6*), in tomatoes was generated for heat tolerance, enhancing fruit setting under heat stress conditions (Klap et al., 2017).

In tomatoes, the *SlMAPK3* gene belongs to the mitogen-activated protein kinase family and participates in response to diverse environmental stress functions. CRISPR/Cas9-mediated genome editing resulted in *slmapk3* mutants showing enhanced thermo-tolerance compared to WT plants and implying its role as a negative regulator of thermo-tolerance (Yu et al., 2019). *BRZ1* positively regulates ROS production in the apoplastic region in tomatoes and serves as a component for heat tolerance. This has been validated from the CRISPR/Cas9-based *bzr1* mutants that showed impaired H_2_O_2_ production in apoplast and heat tolerance by declined Respiratory Burst Oxidase Homolog 1(RBOH1)(Yin et al., 2018). Development of CRISPR/Cas-mediated *HSA1* (heat-stress sensitive albino 1) mutants of tomato showed increased sensitivity to heat stress compared to wild-type plants (Qiu et al., 2018). In maize, CRISPR mutants of the *thermosensitive genic malesterile 5 (TMS5)* gene improved thermosensitive male-sterile plants (Li et al., 2017). In lettuce, the germination of the seeds at a higher temperature was achieved through knockouts of *NCED4*, a key regulatory enzyme in the biosynthesis of ABA. Therefore, mutants of *LsNCED4* could be commercially valuable in production areas with high temperatures (Bertier et al., 2018).

### 4.4 Cold stress tolerance

Low temperature is a key abiotic stressor that adversely influences plant growth and productivity (Jha et al., 2017b). In plants, cold stress tolerance is a highly intricate trait concerning several diverse cell compartments and metabolic pathways (Hannah et al., 2006). Conventional breeding approaches have achieved adequate success in enhancing the cold tolerance of significant crop plants relating to inter-specific or inter-generic hybridization. Cold stress causes damaged seedlings, poor growth, and a low germination rate in rice. It can also decrease grain yield at reproductive phage in rice (Koseki et al., 2010; Shakiba et al., 2017). CRISPR/Cas9 is an attractive and accessible technology for developing non-transgenic genome-edited crop plants to overcome climate change and ensure future food security (Mo et al., 2020). In rice, editing is guided to knockout some negative regulator transcription factors to increase plant tolerance for cold. *OsMYB30* is a transcription factor that binds to the promoter of the β-amylase gene and negatively influences cold tolerance. Under cold stress, *OsMYB30* makes a complex with *OsJAZ9* and inhibits the expression of β-amylase gene, thus affecting starch degradation and maltose accumulation, which may contribute to increasing cold sensitivity (Lv et al., 2017). CRISPR/Cas9-based genome editing of three genes, *OsPIN5b*, *GS3*, and *OsMYB30*, mutated simultaneously and showed enhanced yield and tolerance to cold stress (Zeng et al., 2020). Plant annexins are involved in the regulation of plant development and protection from environmental stresses: Rice annexin genes *OsAnn3 and OsAnn5* are positive regulators of cold stress tolerance at the seedling stage. The Knocking out of *OsAnn3 and OsAnn5* resulted in sensitivity to cold treatments (Shen et al., 2017). In addition, *OsPRP1* enhances cold tolerance in rice by modulating antioxidants and maintaining cross talk through signaling pathways. Knockout of *OsPRP1* induced cold sensitivity in rice, and mutant lines accumulated less antioxidant enzyme activity and lower levels of proline, chlorophyll, ABA, and ascorbic acid (AsA) content relative to WT under low-temperature. Tomato plants are sensitive to chilling stress; therefore, their fruits are more prone to be damaged by cold stress. CRISPR/Cas9-based *cbf1* mutants showed that C-repeat binding factor 1 (*CBF1*) protects the tomato plant against chilling/cold damage and decreases electrolyte leakage (Li et al., 2018b). These plants also showed a higher accumulation of hydrogen peroxide and indole acetic acid, thus, providing tolerance to cold stress in tomato plants. The expression of ten transcription factors from the WRKY family was observed to be two-fold higher under cold stress (Chen et al., 2015). In Cucumber, over-expression of the *CsWRKY6* gene showed enhanced tolerance to cold stress and sensitivity to ABA and proline accumulation (Zhang et al., 2016b). RNA sequencing of *Brassica napus* revealed various genes from the *WRKY* family that play an important role in cold response (Ke et al., 2020). The *SlNPR1* expression and protein content was found to be increased under low temperature (4°C) in tomato. CRISPR/Cas9-mediated genome editing of *SlNPR1* induced the symptoms of chilling injury in tomato plant that was substantiated by the accumulation of hydrogen peroxide (H_2_O_2_), superoxide anion (O2−), and malonic dialdehyde (MDA), and reduction in proline content, antioxidant enzymatic activities, and soluble protein content (Shu et al., 2023).In strawberry, overexpression of *FvICE1* gene resulted in tolerance to cold and drought at the phenotypic and physiological levels. However, CRISPR/cas9-mediated gene edited strawberries mutant showed lower cold and drought tolerance. These results suggested that *FvICE1* functioned as a positively regulator of cold and drought (Han et al., 2023).

### 4.5 Metal stress tolerance

Heavy metal stress is one of the key problems that adversely affect the agricultural productivity of plants (Jha and Bohra, 2016). Plants practice oxidative stress upon contact with heavy metals, leading to cellular injury (Yadav, 2010). Additionally, the accumulation of metal ions in plants perturbs cellular ionic homeostasis. Therefore, plants have developed detoxification mechanisms to reduce heavy metal exposure’s damaging effects and accumulation. Such mechanisms involve controlled elimination of toxic ions from roots, metal uptake, efficient neutralization of metal ions in the protoplast, and appropriation or translocation to remote organs (Sruthi et al., 2017). Various genes direct these mechanisms to enhance tolerance to heavy metal stress (Hasanuzzaman et al., 2019). For example, the loss-of-function mutant of γ-glutamylcyclotransferase showed defensive characteristics against heavy metal toxicity suggesting that the loss-of-function mutants of *OXP1* and γ-glutamylcyclotransferase demonstrate heavy metal and xenobiotic detoxification due to increased glutathione (GSH) accumulation (Paulose et al., 2013). Therefore, developing CRISPR/Cas9-mediated mutants would be useful to fight against the heavy metal stress in plants. Recently, Baeg et al. (Baeg et al., 2021) developed oxp1/CRISPR mutant *Arabidopsis* plants that showed resistance to Cadmium, suggesting an improved capability of heavy metal detoxification in mutant plants compared to WT Col0 plants. Consequently, this study showed a way to confer resistance to xenobiotics and heavy metals in plants by indel mutations using the gene-editing method (Baeg et al., 2021).

In rice, the roots absorb Cadmium from the soil with the transporters *OsNramp1*, *OsNramp5*, and *OsCd1*. *OsHMA3* plays the role of Cadmium sequestration into root vacuole and negatively regulates xylem loading, and *OsLCT1* is involved in Cadmium transport to the grains (Chen et al., 2019a). Manipulating the expression of these transporter genes by genome editing has found some success in reducing Cadmium in the grain crop. The CRISPR/Cas9-based mutants of *OsNramp5 and OsLCT1* genes resulted in allow Cadmium level in rice (Lu et al., 2017; Tang et al., 2017a; Wang et al., 2017b). Similarly, *OsARM1* regulates As-associated transporter genes in rice. It is expressed in the phloem of the vascular bundle in the basal and upper nodes. Knock-out of the *OSARM1* by CRISPR improves tolerance, while its overexpression has increased sensitivity to As (Baeg et al., 2021). Cs+-permeable *OsHAK1* transporter in rice is the major pathway for Cs+ uptake and translocation. To minimize the radioactive caesium (Cs) uptake by rice plants in Fukusima soil contaminated with 137 Cs+, the CRISPR-Cas system was used to obtain transgenic plants lacking *OsHAK1* function. The*OsHAK1*knock-out plants displayed strikingly reduced levels of 137 Cs+ in roots and reduced radioactive caesium contents (Nieves-Cordones et al., 2017). Another instance of using the CRISPR-Cas-based editing in rice is to know the function of a potential target, *OsPRX2*, for improved potassium deficiency tolerance. *OsPRX2* is known to reduce the production of ROS in a K+ limiting condition. It was found overexpression of *OsPRX2* causes the stomatal closing and K+ deficiency tolerance to increase. At the same time, knockout of *OsPRX2* leads to serious defects in leaves phenotype and the stomatal opening under the K+-deficiency tolerance (Mao et al., 2019).

### 4.6 Herbicide tolerance

Weeds are global agricultural constraints that threaten crop production by posing stiff competition for the main crop for nutrients, soil moisture, light, space, and CO2. Weed growth is one of the key factors that influence the quality and yield of crop plants (Sundström et al., 2014). Several approaches have been tried to eradicate weeds (Green, 2014). The herbicide application is the key tool used for weed management in recent crop production systems (Gage et al., 2019). Herbicide tolerance is one of the most important traits of crop plants that advance farming techniques and the productivity of crop plants. CRISPR/Cas9-based genome editing to develop herbicide-resistant crop plants is now the ideal system to control weeds (Toda and Okamoto, 2020). Herbicide-tolerant crop plants showed higher yields and could minimize toxicity to the environment and our body three times compared to crops cultivated through the conventional method(Dong et al., 2021). This should be adapted as an important practice for high-scale farming; cost-effective and requires less effort to develop transgene -free wheat germplasms containing herbicide tolerance mutations that provide tolerance to aryloxyphenoxy propionate-, sulfonylurea-, and imidazolinone-type herbicides by base editing the acetyl-coenzyme A carboxylase and *acetolactate synthase (ALS)* genes (Zhang et al., 2020). *Acetolactate synthase 1* (*ALS1*) is a crop plant’s most important enzyme for herbicide tolerance. CRISPR-mediated genome editing technique has also been applied to introduce herbicide tolerance in crop plants [**Table 3**]. Some examples of crops that have been edited for ALS gene-based herbicide resistance include tomato, soybean, rice, wheat, maize, watermelon, oilseed rape, tabaco, potato and arabidopsis (Dong et al., 2021). Overall, genome editing offers a powerful tool for developing herbicide-resistant crops by modifying key genes such as *ALS*. A new herbicide tolerance trait has been incorporated in *oryza sativa* through CRISPR-based editing of the *OsALS1* gene (Kuang et al., 2020). Mutants of rice generated by developing a new allele (G628W) by G-to-T transversion at 1882 position in the *OsALS* gene showed strong herbicide tolerance. The progenies of rice mutants were transgene-free and harbouring homozygous alleles (G628W) that were agronomically similar to the wilting type. These mutant plants of rice conferred resistance to imazethapyr (IMT) and imazapic (IMP) herbicides (Wang et al., 2021a). Lu et al. (2021) investigated that in rice, new genes and traits can be developed through designing large scale genomic duplication or inversion by CRISPR/Cas9 mediated genome editing. They showed that high transcript accumulation of CP12 and Ubiquitin2 genes were found in leaves and the expression level of HPPD and PPO1 was upregulated by 10 folds in edited plants with homozygous structural variations alleles and resulted in herbicide resistance in field trials without hampering the yield and other agronomically important traits (Lu et al., 2021b). CRISPR/Cas9-mediated genome editing can be useful to generate herbicide-tolerant crop plants. The CRISPR/Cas9-based targeted mutagenesis of three genes ALS (acetolactate synthase), EPSPS (5-Enolpyruvylshikimate-3-phosphate synthase), and pds (phytoene desaturase) conferred herbicide resistance in *Solanum lycopersicum* cv. Micro-Tom (Yang et al., 2022). These herbicide tolerance traits offer a potentially powerful approach to weed management. Thus, the CRISPR-based genome-editing tool could precisely advance the engineering of herbicide-resistant genes in crop plants. However, it is important that the use of such crops must be carefully monitored to prevent the development of herbicide-resistant weeds and other unintended consequences.

### 4.7 Mechanism for abiotic stress tolerance

Genome-editing technology has been considered a significant tool and revolutionized the development of abiotic stress-tolerant crop plants in recent decades. Genome-editing approaches could demonstrate plant tolerance to abiotic stresses by targeting stress-responsive genes, sensitive genes or negatively regulating genes and activating positively regulated genes that control abiotic stress responses. Known positive and negative regulators of a certain trait are ideal targets for crop improvement. Knock out in structural genes, regulated genes, transcription factors and promoter regions produce altered and broken protein products and generate stress tolerant phenotype through modification of biochemical pathways, metabolite profile and physiology. For example, mutagenesis of a structural gene semi-rolled leaf1, 2 in maize confers curled leaf phenotype and drought tolerance by influencing protein expression patterns and ROS scavenging in rice (Liao et al., 2019). In tomatoes, *SlMAPK3* acted as a negative regulator of defense response to heat stress, and the knockout of *SlMAPK3* enhances tolerance to heat stress involving ROS homeostasis (Yu et al., 2019).

**Figure 3:**
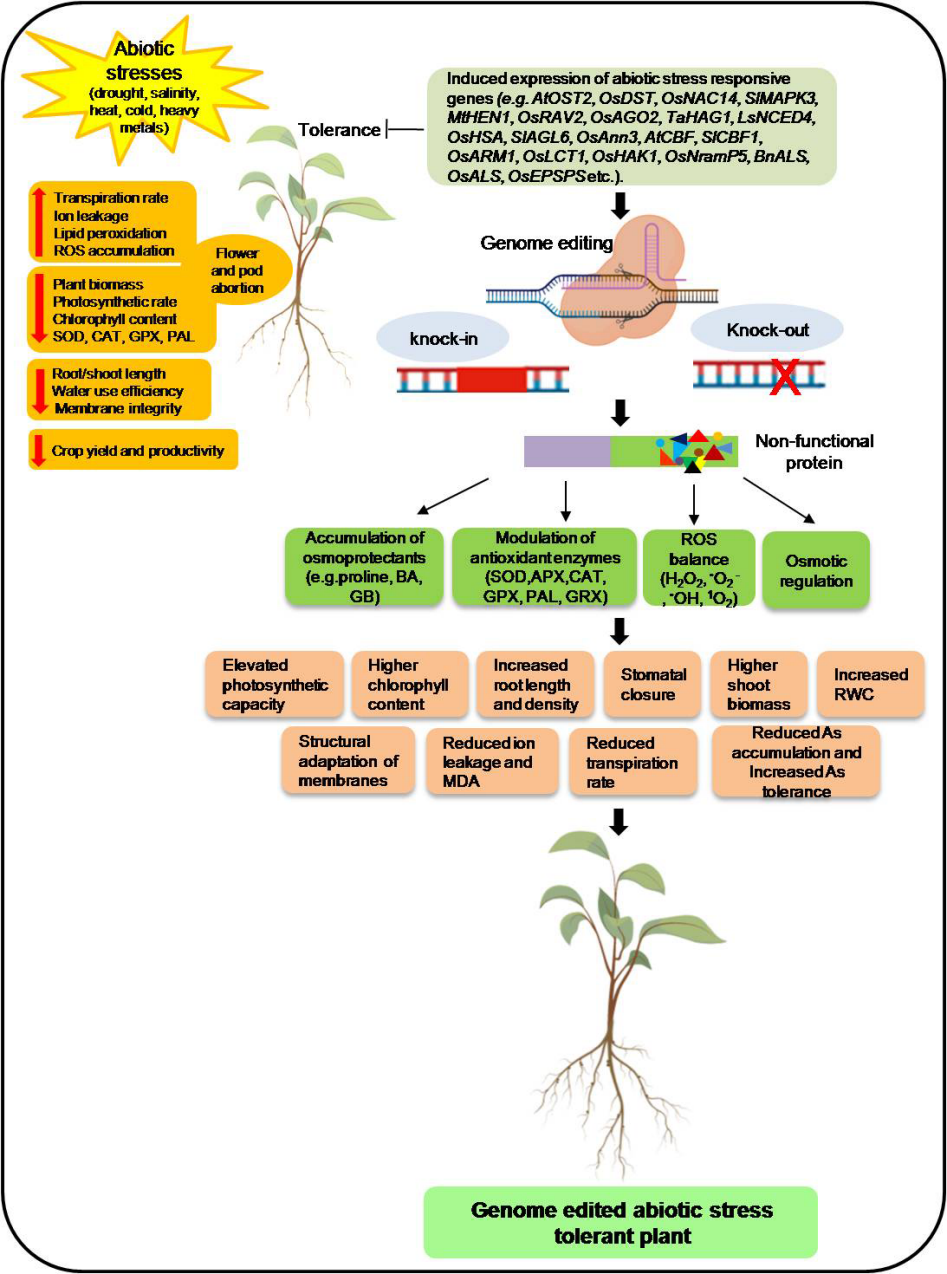
Schematic representation of genome editing mediated abiotic stress (drought, salinity, heat, cold, heavy metals) tolerance in plants. The model shows the stress-induced expression of the abiotic stress-responsive gene that leads to reduced plant biomass; photosynthetic rate; SOD, CAT, GPX, and PAL activities; and chlorophyll content and increased reactive oxygen species (ROS), flower and pod abortion, transpiration rate, ion leakage, and lipid peroxidation. Genome-edited knock-out/knock-in of stress-responsive genes resulted in broken/modified protein that modulates biochemical and physiological characteristics in plants and provides abiotic stress tolerance. SOD, superoxide dismutase; CAT, catalase; GPX, guaiacol peroxidase; PAL, phenylalanine ammonia-lyase; MDA, malondialdehyde; RWC, relative water content; EL, electrolytic leakage, As, Arsenic. Figure created with BioRender.com (https://app.biorender.com/biorender-templates).

Similarly, knock out of the negative regulator of drought tolerance *SlLBD40*, a lateral organ boundaries domain transcription factor, enhances drought tolerance in tomatoes (Liu et al., 2020). **Figure 3** demonstrated these negative regulators of the stress-responsive genes and their inhibitors by editing them to improve stress tolerance for salinity, drought, cold, etc. In addition to the knockout and allele insertion-deletion, the CRISPR editing tool also generates tolerant plants by modulating the genomic aspects through transcript activation, epigenetic modification by chromatin unwinding, and swapping promoter regions with HDR (Li X et al., 2022). These Arabidopsis mutant plants were epigenetically modified for ABA/AREB1 responsive transcription factor by chromatin unwinding, contributing to improving drought tolerance (Roca Paixão et al., 2019). Similarly, the CRISPR/Cas9 transcript activation of *Arabidopsis thaliana* vacuolar H+-pyrophosphatase (AVP1) leads to increased leaf areas and enhanced tolerance to drought stress (Park et al., 2017). CRISPR/Cas9 system has also witnessed the improvement of drought tolerance by creating novel allelic variation in maize using HR-mediated promoter switching (Shi et al., 2017). Maize *ARGOS8* is a negative regulator of ethylene responses. ARGOS8 variants created by replacing its promoter with *GOS2* increased plant yield under drought (Shi et al., 2017). In **Figure 3**, we demonstrated the probable mechanisms of CRISPR/Cas9-based editing of sensitive genes and negative regulators by which plants perceive environmental stresses, transduce their signals and, throughout a sophisticated and finely coordinated response, integrate stress signals, hormonal metabolism and adaptive responses that lead to tolerance. Various structural and regulatory genes (e.g., non-coding RNAs) are known for responding to diverse environmental stresses that can be targeted using CRISPR/Cas9 to improve stress tolerance in agronomically important crop plants (Zafar et al., 2020). Besides developing knockout mutants of individual genes through the CRISPR/Cas system, it can also be used to activate the expression of important candidate genes involved in abiotic stress tolerance.

Additionally, the expression of sensitive genes enhances abiotic stress responses in plants through impaired biochemical (chlorophyll content, changes in antioxidants activities, increased ROS production, ion leakage, lipid peroxidation), physiological (reduced biomass, photosynthetic rate, and higher transpiration rates) and phenotypic (flowers/pods abortion) responses that result in reduced crop yield. Remarkably, CRISPR/Cas9-mediated genome editing approach offers better stress resilience in crop plants through silencing or modification of target genes controlling biochemical, physiological, and morphologicalcomponents. Furthermore, these genetically edited CRISPR plants show elevated photosynthetic capacity, increased root length and density, increased biomass, increased nutrient accessibility, stomatal closure, higher chlorophyll content, reduced transpiration rate, the structural adaptation of membranes, and increased relative water content, decreased electrolytic leakage and malondialdehyde content, the reduced metal accumulation that results in abiotic stresses tolerance in plants **Figure 3**.

## 5 Genome editing driven breeding strategies for abiotic stress tolerance

Genome editing can be integrated into a breeding pipeline for abiotic stress tolerance and gene validation in crop plants. The first step is to identify genes that are known to confer abiotic stress tolerance in plants. This can be done using transcriptomic or genomic data, as well as literature surveys. Once the target genes have been identified, their functions can be validated based on knockout or knock-in mutations induced in the target genes using genome editing. Knockout mutations disrupt the function of a gene, while knock-in mutations introduce a specific trait or function to the target gene. The effect of these mutations can be evaluated by analyzing the resulting phenotypes of the edited plants under different abiotic stress conditions. After validation of the target gene function, the next step is to develop true-breeding lines that carry the desired mutations, which can be obtained by using standard breeding techniques, such as backcrossing or selfing, eventually leading to the generation of stable homozygous lines with the desired mutations. The edited lines can then be tested in the real field conditions challenged by target stress to evaluate their performance =. This is important to ensure that the edited lines are not only tolerant to abiotic stress, but also have high yield and quality traits.

**Figure 4:**
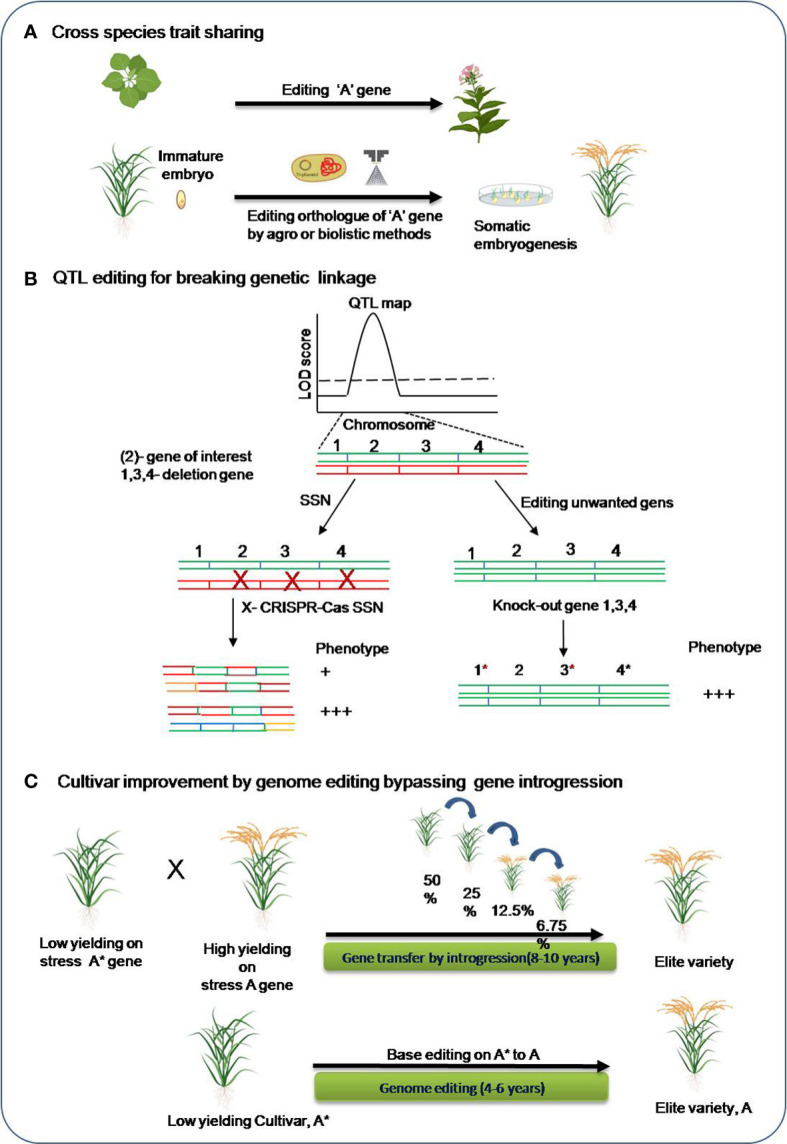
Genome editing in the breeding pipeline for abiotic stress tolerance. **(A)** Trait sharing across species **(B)** QTL editing for high-resolution mapping of the causal genes. SSN: single-stranded nuclease, green and red colored chromosomes are homologous chromosomes. **(C)** Base editing approach for bypassing long-duration introgression breeding for cultivar improvement. *, It is to indicate variety A*.

In plant model organisms, research on genome editing have been carried out for improving tolerance for various abiotic stresses. Moreover, identifying a target gene for editing are much harder in crop species with complex genomes for both forward and reverse genetic studies. However, there are many of the traits such as flowering time, disease resistance, plant height, and seed size that are reported to be conserved across different plant species (Eshed and Lippman, 2019). For example, editing a recessive *MLO* gene in barley resulted in broad spectrum powdery mildew resistance phenotype and similar phenotype was obtained for the editing its orthologous copy in other crops species including wheat (Wang et al., 2014), tomato (Nekrasov et al., 2017) and grapevine(Wan et al., 2020). Hence, trait sharing for complex crop species could also done utilizing the genetic information obtained from the model plant research. Genome editing can also be incorporated in the breeding pipeline aiming to incorporate stress tolerant gene(s) in crop cultivars in a lesser time than the conventional breeding. In most cases of abiotic stress, genetic mapping has found quantitative trait loci (QTLs) in the genome that contain a number of candidate genes. The alternate approach directly edits the unwanted genes, making them nonfunctional and thereby overcoming the deleterious effect of the other genes. Genome editing in breeding pipeline for abiotic stress tolerance in crops is schematically represented in **Figure 4**.

Apart from this, genome editing can assist development of an improved cultivar by-passing traditional introgression. Many important agronomic traits are confirmed to be determined by single-nucleotide polymorphisms, improved crop varieties could be developed by the programmed and precise conversion of targeted single bases in the genomes. Base editors’ tools can be utilized to get specific changes in the genes of elite crop verities that can give rise to an altered functional protein and contribute tolerance. This will save a lot of time and avoid the need for intensive backcrossing for introgression of the causal allele in elite crop varieties.

## 6 Advantages of genome editing approach over breeding and transgenic technologies

The conventional breeding procedure involves crossing between individuals with contrasting phenotypes to introduce useful traits into the final improved product (Saxena et al., 2019; Saxena et al., 2020). Similarly, mutations breeding is carried out to introduce random mutations genome-wide that greatly expands the genomic diversity. However, this procedure requires a lengthy period (8-12 years) as there is a need for repeated backcrossing to the recurrent parent background to ensure the transfer of the desired trait only (Varshney et al., 2021a; Varshney et al., 2021b). Useful genes or traits can also be transferred from other organisms using transgenic breeding, but it involves random integration of the foreign DNA in the genome. Therefore, the resulting transgenic lines will have to undergo the lengthy and costly process of regulatory evaluation before its commercialization. Genome-editing technology has an advantage over these transgenic methods. Genome editing can make small precise changes in a plant’s existing DNA that mimics changes occurring naturally. It can efficiently modify the plant genome for trait improvement and does not require foreign DNA integration. Repeated backcrossing is not required in this case, and transgene-free lines can be ready in less time (2-5 years). Recently, countries such as the USA, China, India, the UK, and many others have allowed genome-edited plants to undergo a different regulatory process than those applied to genetically engineered products. One of the advantages of CRISPR tools over other genome-editing technologies is its potential for multiplexing, the simultaneous editing of multiple target sites (Mali et al., 2013). Genome editing has several advantages over previous technologies, most meaningfully allowing for targeted, single-gene mutation throughout the whole plant genome. The CRISPR technology deals with an easier, more adaptable, and precise form of mutagenesis that enables the transfer of the anticipated trait to progenies (Georges and Ray, 2017). This method can execute mutations to an exact site inside the targeted gene, making the plants’ properties important (Song et al., 2016) as it can be automated to target specific segments of genetic code or edit DNA with better accuracy (Barrangou, 2015).

## 7 Limitation of genome editing for abiotic stress tolerance in crops

Genome editing techniques, such as CRISPR/Cas9, hold great promise for improving abiotic stress tolerance in crops. However, there are also several limitations that need to be addressed. Abiotic stress tolerance is a complex trait that is controlled by a number of genes and pathways. Editing one or a few genes may not be sufficient to achieve the desired level of stress tolerance, and there may be unintended consequences of manipulating the complex regulatory networks involved in stress response (Abdallah et al., 2015). Genome editing techniques can introduce unintended mutations at off-target sites, which can have negative effects on plant growth and development (Zhao and Wolt, 2017). This risk of off-target mutation can be minimized by careful design of the sgRNAs and by using more specific nucleases, such as Cas12a or Cpf1, that have lower off-target effects. Getting the genome editing machinery, such as the CRISPR/Cas9 system, into plant cells can be challenging. Current methods, such as Agrobacterzum-mediated transformation or particle bombardment, can be inefficient and often result in random integration of the transgene Zhang et al., 2021c. Newer methods, such as ribonucleoprotein (RNP) delivery or electroporation, show promise for more efficient and precise delivery of genome editing components. Though varying from country to country, the use of genome editing techniques for crop improvement might attract regulatory approval, which can be a long and costly process. The regulatory status of genome-edited crops varies across countries, and there is a need for a harmonized regulatory framework to ensure the safe and effective use of genome editing in agriculture. Genome editing technologies are subject to patent protection, which can limit their availability and accessibility, especially for small-scale farmers in developing countries.

## 8 Conclusion and future perspectives

CRISPR/Cas9 is considered the method of choice to edit the genome over other genome editing techniques, such as ZFNs and TALENs, for its high efficiency, low cost, and ease of use. It has been used to modify a wide range of plant species to make sequence-specific editing to characterize the function of genes and their ultimate use for trait improvement (Scheben et al., 2017). It can induce editing in many sites in the genome with the use of multiple gRNAs. This is helpful to stack multiple traits in an elite variety (Yin et al., 2017) and target multiple members in multiple gene families (Wang et al., 2019). While the original Cas9 protein from *Streptococcus pyogenes* is widely used for genome editing, researchers have also explored other natural and engineered CRISPR/Cas9 variants to expand the range of applications and increase the specificity and efficiency of the system. The original Cas9 protein from *Streptococcus pyogenes* has been modified to improve its specificity and reduce off-target effects. For example, high fidelity SpCas9 (SpCas9-HF) (Kleinstiver et al., 2016) and enhanced specificity SpCas9 (eSpCas9) (Slaymaker et al., 2015) have been developed by introducing point mutations that reduce non-specific binding to DNACas9 from other bacteria: Cas9 proteins from other bacterial species have been used for genome editing, including *Neisseria meningitidis* (NmCas9) (Hou et al., 2013), *Staphylococcus aureus* (SaCas9) (Kumar et al., 2018), and *Francisella novicida* (FnCas9) (Hirano et al., 2016). These Cas9 proteins are smaller than SpCas9 and may have different target site preferences, which can be useful for certain applications. Not just limited to editing, this system uses site-specific modification in the genome, such as epigenetic changes (Hilton et al., 2015), regulation of gene expression (Tang et al., 2017b) and base editing. This is done with the fusion of the effector protein of ‘dead’ Cas9 (dCas9) protein which is catalytically dead but has DNA binding activity. In this way, the fusion protein is guided to reach specific sites in the genome to do its job (Konermann et al., 2015). The CRISPR interfering system (CRISPRi) can potentially produce effective and precise transcriptional control without editing (Larson et al., 2013). Therefore, this is better than RNAi technology. This is again carried with the binding of SgRNA to dCas9. The binding of the SgRNA to the complementary region blocks the transcriptional elongation by RNA polymerase, expressing the gene without undergoing cell death and damage to genome (Cho et al., 2018). CRISPR/Cas system can also accomplish gene replacement in plants through the targeted integration of specific genes through homology-mediated recombination. Moreover, the CRISPR/Cas can recombine the genome after the DSB in a heterozygous system. This can be used to induce local recombination in the part of the chromosome that does not participate in meiotic recombination, such as the telomeric end and centromeric region, to explore the untapped genetic potential and to narrow down beneficial QTL to the causal allele for precise mapping and gene identification (Lazar et al., 2020; Shlush et al., 2021).

Although significant progress has been made to increase its efficiency and target specificity, furtherinterventions are required to make it an even more powerful tool. A few such areas include introducing the smaller-sized CRISPR system for efficient genome editing. The existing CRISPR/Cas9 is relatively large to pack into viral vectors. Similarly, the requirement of the NGG PAM site for CRISPR cannot address the editing to all locations in the genome. Hence, a multiple PAM site selection will increase the scope of the editing. The transformation rate and editing efficiency using Agrobacterium-based methods are preferred to produce transgenic events; however, not all crops and other plant species respond well to the transformation and regeneration under selection. In addition, to make it transgene-free, the process takes longer to eliminate the transgene by several back-crossings of the plant having the editing.

CRISPR/Cas9 can accelerate plant breeding through changing genomes speedily in a precise and expected way. Due to its simplicity, efficiency, and versatility, CRISPR/Cas9-mediated genome editing has currently become a prevalent technology and has been extensively used to develop resistance in crop plants (Wolter et al., 2019). It can be used for knockout, replacement and insertion of gene that resulted in loss-of-function, knock down or activation mutants, that can lead to development of abiotic/biotic stress-tolerant crop plants (Li Y et al., 2022). Application of multiplex genome editing would open the ways for the development of genome-edited crops engineered for tolerance against multiple traits in a single transformation event. Thus, it is predicted that genome editing will become the technology of choice (Jogam et al., 2022). The lack of validated target for genome editing would be main obstruction in unlocking the CRISPR potential to develop stress tolerant crop plants.

Genome-edited products have the potential to revolutionize many industries, including agriculture and medicine, but they also raise important regulatory and ethical questions. In many countries, genome-edited products are regulated similarly to genetically modified organisms (GMOs), but there is some debate about whether this is the appropriate approach. Looking ahead, it is likely that legal and regulatory frameworks covering genome-edited products will continue to evolve as new products are developed and the technology becomes more widely adopted. It will be important for policymakers to strike a balance between ensuring the safety of these genome-edited products and allowing them for innovation and economic growth. Ultimately, the regulatory frameworks for genome-edited products will need to consider both the potential benefits and risks associated with the technology having unprecedented potential for delivering stress-tolerant crop cultivars.

## Author contributions

Conceptualization, MK; writing—original draft preparation, MK, and MRP; writing—review and editing, MK, MRP, MKP, PKS, AB, BG, and RKV; Funding acquisition, RKV; All authors contributed to the article and approved the submitted version.
